# Supplementary material for: On the temporal spreading of the SARS-CoV-2
Source: PLoS One. 2020 Oct 29;15(10):e0240777. doi: 10.1371/journal.pone.0240777 (PMC7595331; doi:10.1371/journal.pone.0240777)
Supplement: Attachment — Submitted filename: report_PLOS_ONE.pdf [file pone.0240777.s006.pdf]

Review of the paper:

On the temporal spreading of the SARS-CoV-2

Authors:
Francesca Bertacchini, Eleonora Bilotta, Pietro Pantano

In the paper under review the authors analyze the time-space dynamics of the spread of the COVID19.
After the Introduction, in Section 2 they focus on the spatial structure of the virus diffusion. In Section 3 they pass to analyze the temporal evolution of the contagion, while in Section 4 they study the transition phases of the time evolution.

The methods they use are essentially of statistical nature. For this reason I think that the paper under review belongs to the field of statistical modeling rather than mathematical modeling, and I suggest to change the respective keyword.

What follows is a list of comments and requests of clarification that the authors should address

1. The Figure 1 is not clear to me; in particular the author should clarify how they rank the countries; this concept is used several times in the paper.
2. In several instances the authors seem to use the concept of power law, while it seems to me that the data follow an exponential decay law.
3. In Section 4.3 the authors say that the data follows the Fermi-Dirac distribution. To me it seems that the formula they write is a logistic law.
4. In Section 4.4 they say that the data display an oscillatory behavior but I could not find a full support of this statement.
5. The authors say that they have revealed a *chaotic behavior* of the contagion, which I think it is not fully justified. The more generic *complex behavior* seems to me more appropriate.
