# Supplementary material for: On the temporal spreading of the SARS-CoV-2
Source: PLoS One. 2020 Oct 29;15(10):e0240777. doi: 10.1371/journal.pone.0240777 (PMC7595331; doi:10.1371/journal.pone.0240777)
Supplement: Attachment — Submitted filename: Report_PLOSONE_BBP.pdf [file pone.0240777.s007.pdf]

Report on the paper:
On the temporal spreading of the SARS-CoV-2

Authors: F. Bertacchini, E. Bilotta, P.S. Pantano

Most of the mathematical modeling I am familiar with affords the study of the contagion following the spreading of the COVID-19 disease using a dynamical system approach, essentially based on SIR-type models. The paper under review takes an original perspective, with the aim to reveal interesting spatio-temporal features of the disease not emerged so far.

Here it follows a list of comments and questions the authors should address to improve the quality of the presentation:

- Fig. 1(e):
  - The authors write in the caption: 'Population number versus the number of infected on a logarithmic scale': probably they exchange what is plotted on the x-axis with the quantity plotted on the y-axis. More important: Why 'population number'? In the axes of the figure, it is in fact written 'Country Rank Order'. The definition of 'Country Rank Order' is not introduced. The authors should explain how they order the countries and should state precisely what is reported in Fig.1 (e).
    Maybe the countries are in descending order according to the number of infected but this would be in contrast with the fact that the graph reported in fig.1(e) is not monotonously decreasing.

  - Since Fig.1 describes the spatial distribution of the contagion, one should we imagine that the data are taken on a fixed day. This is not specified in the text. On what day is the data used in Figure 1 taken?

  - In the text the authors write: 'Fig. 1(h) plots how the previously obtained EXPONENTIAL function fits real data'. If the authors hypothesize an exponential functional dependence of y on x , why three lines above they write: 'spatial diffusion of COVID-19 follows a POWER LAW'? (see also comment below, Section 4)

  - The caption of Fig.1(e) is probably referred to Fig. 1(h). Please verify and correct the caption of Fig.1

  - How are the nodes of the graph reported in Fig. 1(i) connected? This is not specified in the text nor in the caption of the figure.
- Fig.4:
  - The graph is not clear also in relation to what is written in the text and in the caption. In the graph: 'Log(Day) versus Country Rank Order'. In the text and in the caption: 'The distribution of the number of infected individuals for each country over time'.
    It is unclear what is represented in Fig.4. One could argue that this should be an important point, since the outcomes of the analysis of Fig.4 should be significant arguments to prove the two following claims stated in the Introduction and in the

Conclusions of the manuscript: 1) the virus has scale invariance; 2) propagation in space and time follows a power law.

- Section 4:
  - In the text the authors write: 'If we sort the time differences between the first and the tenth case, we observe that the curve seems to have an EXPONENTIAL trend that therefore can be captured by a straight line on a logarithmic scale, highlighting a POWER LAW'. If one supposes an exponential dependence of y on x, the corresponding graph on a semilogarithmic scale gives a straight line. Namely, if:

    y=A exp(-Bx)   it then follows that

    Log (y) = Log (A) -B x

    Hence, on a semilogarithmic scale (x, Log(y)) one in fact gets a straight line (as, for example, reported Fig.6).
    On the other hand, if one supposes a power law dependence of y on x, one has:

    $$y = \frac{A}{x^B} \qquad B > 0$$

    From which, taking the Log of both sides, it follows:

    Log(y)=Log(A)- B Log(x)

    This last relationship gives a straight line in a (Log(x), Log(y)) graph, which is not the type of graph used in the manuscript. Therefore, why do the authors use the expression 'Power law'?
    In the Zipf distribution, for instance, as it is used in linguistics, the graph usually adopted is (Log(x), Log(y)) and a power law dependence is then found.

- Fig.6:
  - In the caption the authors write: '…for the 44 countries that took a week to reach the tenth case…'. Do they mean: 1) exactly one week (but this would be inconsistent with the data reported in the Tab.); 2) at least one week; 3) not more than a week?
  - Please illustrate in the text and in the caption what is reported in the x- and y-axis of Fig.6 (b): it is not clear.

- Tab.2:
  - See above: the fit of the data is performed on a scale (Log(x), Log(y)) or (x, Log(y))?
  - In the caption of Tab.2 the authors write: 'As it can be noticed, the intervals are invariant along the scale steps'. What does this mean? It is unclear.
- Text below Tab.2:
  - The authors say: 'This may also be related to the fact that the number of samples available decreases with each change of scale'. Why? If one looks at the data presented in tab.1 on would argue that the number of countries for which one

has a transition from 1->10, da 10->100, da 100->1.000 e da 1.000->10.000 is the same. Please explain and clarify.

- Text below Tab.3:
  - The text written in the caption is unclear (the same is also reported in the body of Sez.4.4) starting from: 'The steps between the points of interest…'. Probably in tab.3 are reported, for each country, the differences in days after the hotspot, necessary to pass from one temporal scale to the next one. However this should be specified in the caption of Tab.3. Please clarify the meaning of the second phrase written in the caption.
- Caption of Fig.12:
  - Why do the authors write: 'clustering of THREE pairs of intervals..'? All the pairs of time intervals are reported in the histogram or only three pairs?
- Sez.4.4 after Fig.13:
  - b. Pandemic Outbreak (text written referring to Fig.13). The authors write: 'Even in this phase the virus reaches critical phases, also presenting an oscillatory behaviour'. What do the authors mean? From which data/graph one can derive an oscillatory behavior? Do the authors mean oscillations in time or varying the country at a fixed time? In this second case, by inspection of Fig.13, the dependence of the outbreak time vs country does not seem to have definite oscillations.
- Tab.6:
  - Referring to Tab.6 (and, therefore, to the data that allow to aggregate different countries on the basis of the evolution of the contagion), it would probably be appropriate that in the analysis the authors would take into account the differences among countries regarding the geography, density of population, social habits, etc. One can image, for instance, that the data of the spreading of the contagion are strongly influenced by density of the population. One should therefore perform the analysis within classes of countries of comparable density of population. Besides, it would probably be more significant to consider, instead of the absolute number of infected individuals for each country, the fraction of infected with respect to the total population.
- Conclusions:
  - The phrase: '…we then realized that each country is achieving not only a rate of evolution at the world level, but also at the level of individuals regions or provinces' is unclear. On the basis of which analysis/graph one could compare the global (at the world level) behavior and the behavior at the level of individual regions or provinces. The authors should clarify which data/analysis support this thesis.
