# Supplementary material for: On the temporal spreading of the SARS-CoV-2
Source: PLoS One. 2020 Oct 29;15(10):e0240777. doi: 10.1371/journal.pone.0240777 (PMC7595331; doi:10.1371/journal.pone.0240777)
Supplement: Attachment — Submitted filename: Response to Reviewers.docx [file pone.0240777.s008.docx]

# **Responses to Reviewers**

First, we would like to thank the Editor and the Reviewers who have given a valuable contribution to the improvement of this work.

After completion of the suggested edits, the revised manuscript has benefitted from an improvement in the overall presentation and clarity.

As general remarks, we would like to say that we accept the Reviewer's suggestion to change the key word “SARS-CoV2 mathematical modelling” to “SARS-CoV2 modelling”.

We have also removed the highlights because they are not included in the final formatting.

We have added titles to the tables.

Below, you will find a point-by-point description of how each comment was addressed in the manuscript. Original Reviewers’ comments are in boldface, responses in regular typeface.

**Reviewer 1**

1. **The Figure 1 is not clear to me; in particular, the author should clarify how they rank the countries; this concept is used several times in the paper.**

In order to respond to the Reviewer's request and to clarify the concept of country rank order, we have introduced the following sentence in the main text: “If we order all the countries according to the number of infected and number them progressively from 1 to 217, we get the infected country rank order”.

2. **In several instances, the authors seem to use the concept of power law, while it seems to me that the data follow an exponential decay law.**

As for Reviewer's suggestion on power law, we have corrected all the points mentioned in the main text that refer to power law, replacing them in some cases with the terms exponential law.

3. **In Section 4. 3 the authors say that the data follows the Fermi-Dirac distribution. To me it seems that the formula they write is a logistic law.**

We accept the Reviewer's suggestion and inserted Logistic function instead of Fermi-Distribution Function.

4. **In Section 4.4 they say that the data display an oscillatory behaviour but I could not find a full support of this statement.**
5. We accept the Reviewer's proposal by deleting the sentence about oscillatory behaviour in the first case.
6. **The authors say that they have revealed a chaotic behaviour of the contagion, which I think it is not fully justified. The more generic complex behaviour seems to me more appropriate.**

In the second sentence in which we cite the oscillatory behaviour, we have changed defining the phenomena under examination as complex behaviour.

**Reviewer 2**

1. **• Fig. 1(e):**
   - **The authors write in the caption: ‘Population number versus the number of infected on a logarithmic scale’: probably they exchange what is plotted on the x-axis with the quantity plotted on the y-axis. More important: Why ‘population number’? In the axes of the figure, it is in fact written ‘Country Rank Order’. The definition of ‘Country Rank Order’ is not introduced. The authors should explain how they order the countries and should state precisely what is reported in Fig.1 (e). Maybe the countries are in descending order according to the number of infected but this would be in contrast with the fact that the graph reported in fig. 1(e) is not monotonously decreasing.**

We have changed the caption for Fig. 1 *e* with Fig.1 *h* and vice versa. Regarding the Country Rank order, we have added a sentence to introduce this concept in the main text (as suggested also by Reviewer 1). The sentence is as follows: “If we order all the countries according to the number of infected and number them progressively from 1 to 217, we get the infected country rank order”.

- **Since Fig. 1 describes the spatial distribution of the contagion, one should we imagine that the data are taken on a fixed day. This is not specified in the text. On what day is the data used in Fig.1 taken?**

The starting day of data collection is January 22, 2020. This information is included in the main text. We have added the beginning and the closing of the data collection for this work in the caption of Fig.1 as well. The sentence is the following: “The data collection began on January 22, 2020 and ended on May 28, 2020”.

- **In the text, the authors write: ‘Fig. 1(h) plots how the previously obtained EXPONENTIAL function fits real data’. If the authors hypothesize an exponential functional dependence of y on x , why three lines above they write: ‘spatial diffusion of COVID-19 follows a POWER LAW’? (see also comment below, Section 4)**

We have corrected all the points mentioned in the main text that refer to power law, replacing them in some cases with the terms exponential law. Reviewer 1 has also provided the same suggestions for correction. Therefore, changes have already been made throughout the text.

- **The caption of Fig.1(e) is probably referred to Fig. 1(h). Please verify and correct the caption of Fig.1**

We have changed the caption for Fig. 1 *e* with Fig.1 *h* and vice versa.

- **How are the nodes of the graph reported in Fig. 1(i) connected? This is not specified in the text nor in the caption of the figure.**

To answer the question about how the nodes are connected in the countries graph, we added the following sentence in the caption of Fig. 1. “In the graph, the nodes represent countries while the links connect geographically neighbouring countries”.

2. **Fig.4:**
   - **The graph is not clear also in relation to what is written in the text and in the caption. In the graph: ‘Log(Day) versus Country Rank Order’. In the text and in the caption: ‘The distribution of the number of infected individuals for each country over time’. It is unclear what is represented in Fig.4. One could argue that this should be an important point, since the outcomes of the analysis of Fig.4 should be significant arguments to prove the two following claims stated in the Introduction and in the Conclusions of the manuscript: 1) the virus has scale invariance; 2) propagation in space and time follows a power law.**

We accept the suggestions of Reviewer 2, adding the following sentence: "Starting from the day of the beginning of the pandemic for each country, we have placed all the countries on the x axis. Then, we assigned a number to each country and rearranged the Country Rank Order according to the time parameter".

3. **Section 4:**

**In the text the authors write: ‘If we sort the time differences between the first and the tenth case, we observe that the curve seems to have an EXPONENTIAL trend that therefore can be captured by a straight line on a logarithmic scale, highlighting a POWER LAW’. If one supposes an exponential dependence of y on x, the corresponding graph on a semilogarithmic scale gives a straight line. Namely, if:**

**y=A exp(-Bx) it then follows that**

**Log (y) = Log (A) -B x**

**Hence, on a semilogarithmic scale (x, Log(y) ) one in fact gets a straight line (as, for example, reported Fig.6). On the other hand, if one supposes a power law dependence of y on x, one has:**

**From which, taking the Log of both sides, it follows:**

**Log(y)=Log(A)- B Log(x)**

**This last relationship gives a straight line in a (Log(x), Log(y)) graph, which is not the type of graph used in the manuscript. Therefore, why do the authors use the expression ‘Power law’? In the Zipf distribution, for instance, as it is used in linguistics, the graph usually adopted is (Log(x), Log(y)) and a power law dependence is then found.**

We accept the Reviewer's suggestion and remove all references to power law, as also requested by Reviewer 1. Furthermore, the figure compares the distribution histograms considering the real data (with full rectangles) and those obtained from a Zipf distribution (with points).

4. **Fig.6:**

- **In the caption the authors write: ‘…for the 44 countries that took a week to reach the tenth case…’. Do they mean: 1) exactly one week (but this would be inconsistent with the data reported in the Tab.); 2) at least one week; 3) not more than a week? o Please illustrate in the text and in the caption what is reported in the x- and y-axis of Fig.6 (b): it is not clear.**

As noted by the reviewer, the caption in Figure 6a is misleading. Therefore, we have replaced to "Country Rank Order" the label "Interval Rank Order", replacing in place of $I_{n}$ the symbol$\Delta_{n}$. The final caption is as follows: “**Fig 6. Time differences between the first and the tenth case for the countries**. If we order all the intervals $i$ according to $\Delta_{i}$ values and number them progressively from 1 to 217, we get the interval rank order, curve fitting $3.548\text{ }-0.013x$ (a)*.* Furthermore, the figure compares the distribution histograms considering the real data (with full rectangles) and those obtained from a Zipf distribution (with points) (b).”

5. **Tab.2:**

- **See above: the fit of the data is performed on a scale (Log(x), Log(y)) or (x, Log(y))? o In the caption of Tab.2 the authors write: ‘As it can be noticed, the intervals are invariant along the scale steps’. What does this mean? It is unclear.**

To reply to the Reviewer’s suggestion, we have added the following sentence: “The data fit is performed on a scale (x, Log(y))”.

Since the sentences indicated by the Reviewer may seem ambiguous, we preferred to remove the sentences that give rise to this ambiguity.

6. **Text below Tab.2:**

- **The authors say: ‘This may also be related to the fact that the number of samples available decreases with each change of scale’. Why? If one looks at the data presented in tab.1 on would argue that the number of countries for which one has a transition from 1->10, da 10->100, da 100->1.000 e da 1.000->10.000 is the same. Please explain and clarify.**

Here too we have removed the text “**This may also be related to the fact that the number of samples available decreases with each change of scale”** to make the discussion clearer.

7. **Text below Tab.3:**

- **The text written in the caption is unclear (the same is also reported in the body of Sez.4.4) starting from: ‘The steps between the points of interest…’. Probably in tab.3 are reported, for each country, the differences in days after the hotspot, necessary to pass from one temporal scale to the next one. However this should be specified in the caption of Tab.3. Please clarify the meaning of the second phrase written in the caption.**

We have changed the caption of Tab.3 in the following way. Instead of talking about temporal points of interest in the evolution of the virus, it is better to talk about particularly significant dates (or days).

8. **Caption of Fig.12:**

- **Why do the authors write: ‘clustering of THREE pairs of intervals..’? All the pairs of time intervals are reported in the histogram or only three pairs?**

We have eliminated the term **THREE**, following the Reviewer's proposal.

9. **Sez.4.4 after Fig.13:**

- **b. Pandemic Outbreak (text written referring to Fig.13). The authors write: ‘Even in this phase the virus reaches critical phases, also presenting an oscillatory behaviour’. What do the authors mean? From which data/graph one can derive an oscillatory behaviour? Do the authors mean oscillations in time or varying the country at a fixed time? In this second case, by inspection of Fig.13, the dependence of the outbreak time vs country does not seem to have definite oscillations.**

We have removed the sentence concerning the oscillatory behaviour of the virus, following the suggestion of Reviewer 2. Reviewer 1 had also highlighted this critical issue.

10. **Tab.6:**

- **Referring to Tab.6 (and, therefore, to the data that allow to aggregate different countries on the basis of the evolution of the contagion), it would probably be appropriate that in the analysis the authors would take into account the differences among countries regarding the geography, density of population, social habits, etc. One can image, for instance, that the data of the spreading of the contagion are strongly influenced by density of the population. One should therefore perform the analysis within classes of countries of comparable density of population. Besides, it would probably be more significant to consider, instead of the absolute number of infected individuals for each country, the fraction of infected with respect to the total population.**

What Reviewer 2 suggested would be highly recommended, but since the data are grouped by country, it is not clear what could be the factors that determine the similarities between these growth rates. The issues proposed could be the subject of future developments of this work.

11. **• Conclusions:**

- **The phrase: ‘…we then realized that each country is achieving not only a rate of evolution at the world level, but also at the level of individuals regions or provinces’ is unclear. Based on which analysis/graph one could compare the global (at the world level) behaviour and the behaviour at the level of individual regions or provinces. The authors should clarify which data/analysis support this thesis.**

We are also working on the spatial scale and therefore many data confirming these hypotheses are going to appear in another paper. To give some indications, we can change the sentence by saying that at the time of the analysis of the temporal trend of the virus, we had hypothesized these tendencies described in the conclusions.
